# 306 Review of CRRT in Burn Patients with Non-survivable Injuries

**DOI:** 10.1093/jbcr/irad045.281

**Published:** 2023-08-29

**Authors:** Amanda D Brock, Rebecca Coffey, Samuel P Mandell

**Affiliations:** Parkland Hospital, Kemp, Texas; Parkland Health, Keller, Texas; Parkland Burn Center / UTSouthwestern, Dallas, Texas

## Abstract

**Introduction:**

There is a subset of burn patients that have a non-survivable injury. Careful and thoughtful use of health care resources in this population is needed to provide end of life care. The purpose of this quality improvement project was to review patients with non-survivable injuries (NSI), started on continuous renal replacement (CRRT), to identify areas of improvement in end-of-life care.

**Methods:**

A retrospective review of all patients admitted to one verified burn center from 1/1/2020 – 5/9/2022 who received CRRT as a life prolonging measure in patients with NSI. NSI was defined as modified BAUX predicted mortality of >90 0 and/or significant comorbidities, peri burn events ( i.e. Delayed presentation, cardiac arrest in the field) and physician judgement as documented in the chart. Data points collected include, age, TBSA and BAUX scores, indication for CRRT, any documented goals of care discussions, duration of CRRT, days from admit to initiation of CRRT and additional blood products and CRRT filters.

**Results:**

A total of 21 patients who received CRRT were reviewed. Of those, 1 survived, 2 had incorrect MRNs in the hospital database, leaving 17 patients who died for analysis. The median age was 68 (IQR 18). The median TBSA was 43% (IQR 42). There were 2 patients with 0 % TBSA, 1 sustained a lightning strike and 1 with inhalation only. The median BAUX was 112 (IQR 37). The median hospital Length of stay (LOS) was 12 days (IQR 24). The median time in days to start of CRRT from time of injury was 2.5 days (IQR 11.5). Median time of CRRT initiation to time of death was 48 hours (IQR 239.5 range of 2.5 hours to 720 hours.). Blood product and filter use related to the initiation of CRRT for the 17 patients is in Table 1.

Of the patients included in the review, there were several issues related to the initiation of CRRT. One had a DNR, but family did not provide documentation and/or follow patient’s wishes. Several families were offered CRRT as an option despite futility and then insisted on that progression of care. Others were started on CRRT to allow time for family to be located or to arrive before transitioning to comfort care.

**Conclusions:**

In patients with NSI, CRRT was often initiated within 24 hours of death. In several cases, when comfort measure only was the known plan of care. This in turn led to increased use of blood products and filters. For patients with non-survivable injuries, establishing early goals of care and discussing end of life care is imperative.

**Applicability of Research to Practice:**

Early goals of care conversations and thoughtful use of resources in patients with non-survivable injuries should be examined to determine the appropriateness of care offered and interventions provided.

